# Organellar stress intersects the astrocyte endoplasmic reticulum, mitochondria and nucleolus in HIV associated neurodegeneration

**DOI:** 10.1038/s41419-018-0341-3

**Published:** 2018-02-22

**Authors:** Shruthi Nooka, Anuja Ghorpade

**Affiliations:** Department of Microbiology, Immunology and Genetics, University of North Texas Health Science Center, Fort Worth, TX USA

Human immunodeficiency virus (HIV)-1 infection is no longer a death sentence given the success of antiretroviral (ARV) therapy (ART). HIV-1 invades the central nervous system (CNS) early in infection and over the long run causes neurodegeneration. Despite the dramatic reductions in the severity of these neurocognitive effects in the post-ART era, mild to moderate motor and cognitive impairments persist in HIV-infected individuals that are collectively termed HIV-associated neurocognitive disorders (HAND). While we await the HIV cure, effective adjunctive therapies are needed to target indirect pathological mechanisms of HIV infection that persists in the CNS, especially for cells that are silent for virus production and thus resistant to ART. HIV hijacks the host intracellular and intercellular communication highways, exacerbating neuropathogenesis. Persistent low-level inflammation and oxidative stress contribute to pathological damage in many neurological disorders, including those associated with HIV. One of the principal CNS cell types mediating these events are astrocytes. Thus, further investigations uncovering novel HIV-induced neurotoxic mechanisms in astrocytes and their contributions to HAND are timely.

Eukaryotic cells contain several intracellular membrane-bound organelles with specialized functions. An ongoing dialog between the endoplasmic reticulum (ER), mitochondria, nucleus, Golgi apparatus and others is pivotal for cellular function in both homeostasis and disease. Given that ultimately all human diseases have a cellular and/or molecular basis, cellular stress responses and dialog play key mechanistic roles. Emerging evidence emphasizes organelle communication linking to human pathologies^1^. The challenge is to decipher the multifaceted signaling network of crosstalk among these cellular compartments leading to malfunction and possibly death, which are necessary for identifying specific therapeutic approaches for neurodegenerative diseases.

The mechanisms underlying neurodegenerative disorders are multifactorial and include cellular insults such as oxidative stress, neuroinflammation, mitochondrial dysfunction, excitotoxicity, and accumulation of misfolded proteins^2^. To cope with such stress, cells have established an extensive range of complex response mechanisms that act at the organelle level. In the ER, the unfolded protein response (UPR) is generally initiated by physiological and pathological insults such as high protein demand, viral infections, inflammation, and overload of mutant proteins. Over the last decade, UPR has been implicated in many neurodegenerative diseases^3^. More recently, UPR served as a therapeutic target for treatment and prevention of neurodegeneration by inhibiting the function of ER stress-specific mediators^4^. However, the effect of HIV infection on UPR in general, and particularly in astrocytes, has scarcely been investigated. A significant increase in the ER chaperone BiP, binding immunoglobulin protein expression was upregulated in the gray matter of mid-frontal cortex of patients with HIV infection^5^. Our data revealed that HIV-associated inflammation significantly increased expression of the ER stress markers BiP and CCAAT-enhancer-binding protein homologous protein (CHOP)^6^. We also showed activation of all three UPR pathways in human astrocytes in response to inflammation, thus highlighting mutual regulation between ER-mediated UPR and inflammation during HIV-1 CNS infection^6^.

The neurotoxic side effects of ARV drugs are among several contributing factors to the continued prevalence of HAND^7^. However, ARV drug-mediated toxicity in CNS, particularly in glial cells, remains largely unexplored. Our current data provide initial evidence that ARV drugs, specifically nucleoside reverse transcriptase inhibitors (NRTIs), such as abacavir, trigger astrocyte ER stress responses^6^. This knowledge will help guide the development of treatment strategies aimed at reducing antiretroviral neurotoxicity.

Recent findings revealed that the junction between the ER and the mitochondria plays a critical role in cell death regulation^8^. Increased oxidative stress and calcium dysregulation involving the mitochondria and ER are pivotal in neuropathogenesis^9^. HIV proteins gp120 and Tat disrupt calcium-regulating systems in the plasma membrane and ER in neurons. Disturbances in calcium homeostasis induce injury and/or death in neurons and glia that ultimately result in tissue loss in HIV-vulnerable brain regions. Calcium dysregulation is also implicated in long-term neurodegenerative disorders including Alzheimer’s, Parkinson’s, and Huntington’s diseases^10^.

The ER serves as the main storage organelle for calcium. Our studies showed that a prototypical neuroinflammatory cytokine interleukin (IL)-1β and abacavir induced intracellular calcium signaling in astrocytes and upregulated calnexin expression, a calcium-dependent ER chaperone^6^. Chronic elevated cytosolic calcium in astrocytes can be toxic causing neuronal injury or death. Intracellular calcium chelation reduced HIV-associated astrocyte ER stress, indicating a mechanistic role for calcium dysregulation^6^. Since excessively increased astrocyte cytosolic calcium can be toxic and cause neuronal injury and death, our data suggest that both abacavir and neuroinflammation potentially play a role in development of HAND during long-term ART treatment in HIV+ individuals. Mitochondrial dysfunction is also linked to both HIV and ART. Among ARV drugs, NRTIs are known to induce mitochondrial dysfunction by interfering with mitochondrial DNA replication. Since the ER and mitochondria interact closely with each other, sustained ER stress and calcium release can induce membrane permeability transition pore (mPTP) opening on mitochondria^11^. Mitochondrial dysfunction plays a significant role in several neurodegenerative diseases^12^. In our studies, intracellular calcium chelation significantly reduced IL-1β and ARV-dependent mPTP opening: clearly linking calcium-mediated ER stress to mitochondrial dysfunction^6^.

**Fig. 1:**
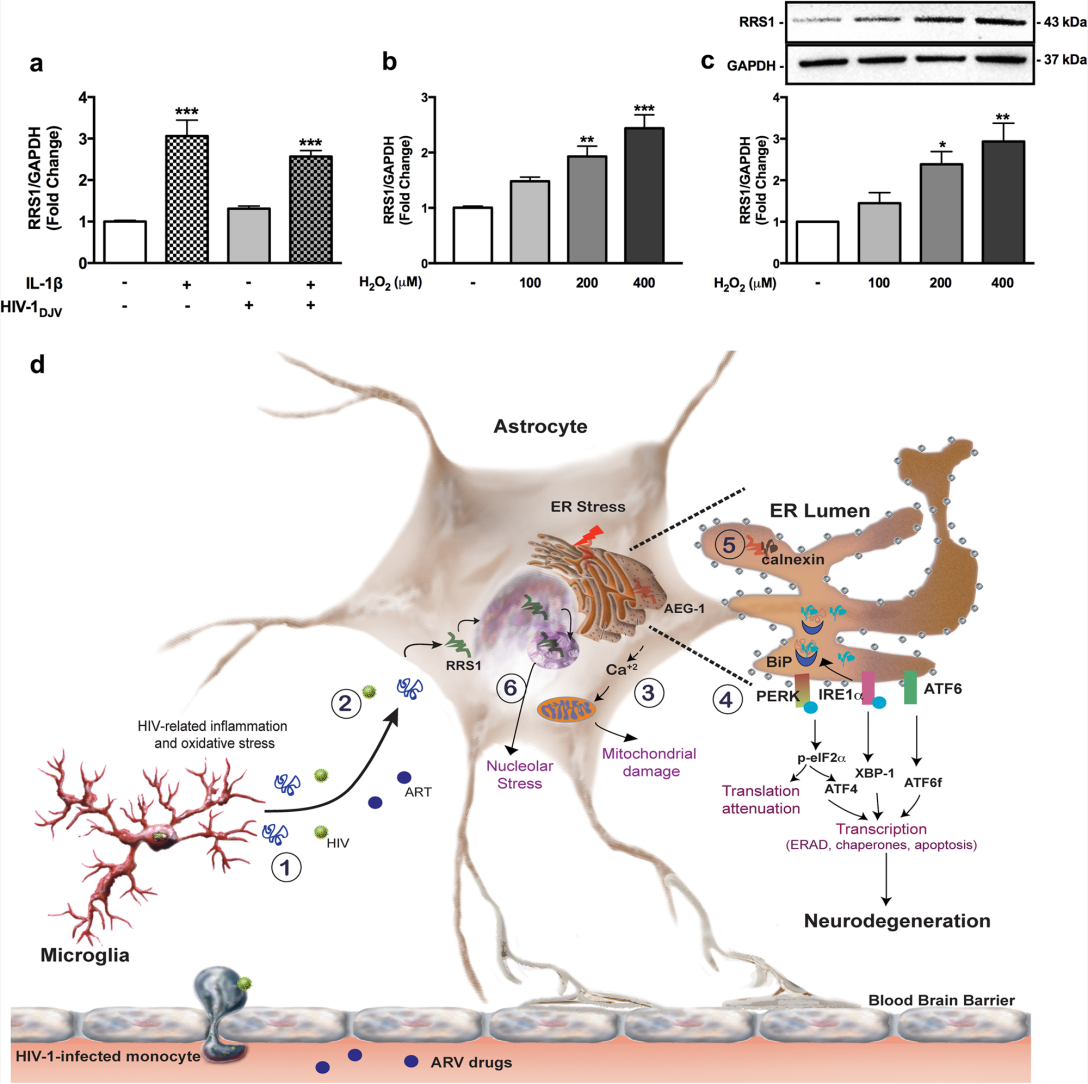
Inflammation and oxidative stress induce astrocyte ER and nucleolar stress leading to mitochondrial dysfunction. Primary human astrocytes were treated with IL-1β (20 ng/ml) and HIV-1 (p24, 20 ng/ml) alone or in combination (**a**) or with increasing concentrations of hydrogen peroxide (H_2_O_2_) (100 μM, 200 μM, and 400 μM) (**b, c**) and untreated astrocytes were maintained in parallel. RNA was isolated (8 h) and RRS1 mRNA levels were analyzed by RT^2^-PCR. Total cell lysates were immunoblotted for RRS1 and GAPDH following 24 h. GAPDH was used as housekeeping and loading controls. A representative blot is presented, while the average fold change from multiple independent donors is graphed. Statistical analyses were performed using one-way ANOVA with Tukey’s post-test for multiple comparisons (*p < 0.05, **p < 0.01, ***p < 0.001). Data represent mean ± standard error of the mean. **d** Astrocyte organelle stress responses during HAND (1) HIV-1 infected/activated CNS cells release HIV-1 proteins, ROS, and proinflammatory cytokines such as TNF-α and IL-1β. (2) Astrocytes become reactive in response to HIV-relevant stimuli. (3) Our data establish that IL-1β- and ARV-mediated increases in intracellular calcium initiate ER stress and mitochondrial dysfunction. (4) Activation of the UPR pathways PERK, IRE1α, and ATF6 result in expression of ER chaperones BiP and calnexin. (5) AEG-1 interaction with the calcium-binding chaperone calnexin suggests a role in calcium signaling. (6) HIV-1-relevant inflammation and oxidative stress significantly increase RRS1 expression, suggesting inhibition of rRNA transcription and nucleolar stress in astrocytes during HIV-1 infection. Taken together, we propose that the organelle network with interlinked stress responses may play a crucial role in HIV-associated neuropathogenesis and could be promising therapeutic targets in HAND. *Scheme abbreviations:* AEG-1 astrocyte elevated gene, ARV antiretroviral, ATF activating transcription factor, BiP binding immunoglobulin protein, Ca^2+^ calcium, ER endoplasmic reticulum, ERAD ER-associated degradation, f fragment, HIV human immunodeficiency virus, ROS reactive oxygen species, p-eIF phosphorylated-eukaryotic initiation factor, PERK protein kinase RNA-like ER kinase, IRE inositol-requiring enzyme, RRS regulator of ribosome synthesis, XBP x-box binding protein

Nucleolar stress, a novel and relatively less studied component of neurodegenerative processes, is associated with impaired rRNA transcription^13^. HIV-1-mediated nucleolar stress is not well documented. Cells combat ER stress, regardless of its source, by inhibiting ribosome synthesis and attenuating protein translation. Since oxidative stress is also implicated in neurodegenerative diseases^14^, we assayed regulator of ribosome synthesis 1 (RRS1) levels in response to inflammation and hydrogen peroxide (H_2_O_2_) (Fig. 1a–c). As a protein that inhibits transcription of both rRNA and ribosomal protein genes, elevated expression of RRS1 indicates that HIV-relevant stimuli augment nucleolar stress in astrocytes.

Astrocyte elevated gene (AEG)-1 was identified as a novel modulator of astrocyte responses to injury and HIV-associated neuroinflammation, signifying central regulatory role for AEG-1 in HAND. In addition, AEG-1 translocated to nucleolus in response to injury and oxidative stress^15^. In the context of our previously published works, we postulated that AEG-1 likely contributes to HIV-1/ARV-induced ER stress in astrocytes. We confirmed AEG-1 as an ER stress inducible gene^6^. To the best of our knowledge, we are the first to report interaction between AEG-1 and calnexin, a calcium-binding chaperone. This comes as no surprise though, as AEG-1 is a multifunctional scaffolding protein. It is enticing to anticipate that AEG-1 also regulates ER-dependent calcium signaling through calnexin.

Together these data indicate that organellar stress is intertwined between the ER, mitochondria, and nucleolus in astrocytes to mediate HIV-associated neurodegeneration (Fig. 1d). HIV, inflammation, and ART elevate intracellular calcium to activate the UPR and mitochondrial dysfunction in astrocytes. HIV-associated inflammation and oxidative stress also initiate nucleolar stress to mitigate protein overload and ER stress in astrocytes during HIV-1 CNS infection. Our work also revealed that AEG-1 likely occupies a unique niche in HIV-associated organellar stress, signifying its contribution towards neurodegeneration. AEG-1 functional range is connected to localization to various subcellular compartments including the nucleus, nucleolus, and ER, making AEG-1 a wide-ranging therapeutic target that mediates astrocyte responses. Taken together, this study provides a framework for further elucidation of intersecting organellar stress responses in HIV-1 neuropathogenesis in order to develop novel therapeutic targets for HAND. Given that these works focus on astrocytes with a central role in all CNS injuries, they shed unique light on the web of organellar stress pathways and have far-reaching implications to neurodegeneration beyond that associated with HIV.
